# The use of borderline personality disorder severity index-iv feedback in adjusting borderline personality disorder treatment: therapists and patients perspectives

**DOI:** 10.1186/s12888-022-04104-w

**Published:** 2022-07-14

**Authors:** Odette de Wilde Brand, Sharon Clarke, Arnoud Arntz

**Affiliations:** 1 De Viersprong, Netherlands Institute of Personality Disorders, PO Box 7, 4660 AA Halsteren, The Netherlands; 2 Department of Clinical Psychology, University of Amsterdam, PO Box 15933, 1001NK Amsterdam, The Netherlands

**Keywords:** Borderline personality disorder, Borderline personality disorder severity index-IV, Routine outcome monitoring, Mentalization based treatment

## Abstract

**Background:**

Previous research has emphasized the importance of therapists giving Routine Outcome Monitoring (ROM) feedback to their patients. It has been shown that several factors influence therapists’ tendency to provide ROM feedback to their patients.

**Methods:**

In this qualitative study, using a semi-structured interview followed by thematic analysis using Atlas.ti, we focused on experiences of therapists and patients with a disorder specific ROM instrument: the Borderline Personality Disorder Severity Index-IV (BPDSI-IV). Ten patients with a borderline personality disorder who had been in Mentalization Based Treatment (MBT) and ten MBT-therapists treating patients with a borderline personality disorder were interviewed.

**Results:**

Qualitative analysis revealed that patients experienced benefits of ROM using the BPDSI-IV. Patients gained more insight in and recognition of their borderline personality disorder symptoms. They also felt more understood by the therapist because they got an opportunity to explain their symptoms in a different way than in a regular therapy session. Therapists shared they didn’t always use all the ROM outcomes as serious feedback for adjusting treatment. They preferred to use the BPDSI-IV over the other ROM instruments, because the BPDSI-IV is disorder specific, which gives insight into the treatment course of the patient.

**Conclusions:**

Experiences of both patients and therapists with the BPDSI-IV were positive. It seems to be valuable and promising for healthcare institutions to evaluate treatment with a disorder specific ROM instrument.

**Supplementary Information:**

The online version contains supplementary material available at 10.1186/s12888-022-04104-w.

## Background

From all patients in therapy, only 50%-75% benefits from treatment [2]. It is unlikely after 1 or 2 years of treatment without progress that continuing treatment will lead to improvement, in many cases however treatment is not terminated nor another treatment is offered.

There are several possible reasons for not ending therapy while no progress is being made: i.e. patients are afraid to deteriorate; patients are feeling supported by their therapist; therapists do not want to disappoint their patients; therapists want to achieve the best result as possible; therapists do not want to admit that treatment is not bringing enough positive change [2].

The accuracy of therapists’ judgment for predicting if patients will deteriorate in treatment seems to be less accurate than the accuracy of a routine outcome instrument. In a study of Hannan et al. [17] therapists predicted the treatment outcome of 550 patients who were seen for individual therapy, couples therapy and crisis interventions. Therapists predicted that three out of 550 (0.01%) patients would deteriorate. At the end of therapy, outcome data concluded that 40 (7.3%) patients deteriorated. The prediction of therapists thus differed from the prediction of an empirical instrument. In the same study [17] patients receiving psychotherapy completed the Dutch Version of the Outcome Questionnaire, OQ-45 [18], in advance of each therapy session. 36 of 492 (7.3%) patients who started treatment, had deteriorated at the end of treatment (average 4.44 sessions). Using the OQ-45as an empirical instrument, 86% of the patients who deteriorated could be predicted at the third therapy session.

Timely ending unsuccessful treatment will reduce waiting lists and high costs and gives patients a chance to enroll in a potentially more effective treatment [2]. To help therapists to objectively track the course of treatment, therapists can use Routine Outcome Monitoring (ROM) [2]. Structurally evaluating patients’ treatment progress gives therapists a chance to adjust therapy when the patient is not on track [22]. And next to that, providing patients feedback on their treatment response can enhance shared decision making [6], which will not only lead to a higher treatment effect but also to better communication between patient and therapist and empowerment of the patient [21].

Feedback on treatment progress is most effective when it is given to both therapist and patient [8]. Unfortunately therapists do not always use ROM feedback in this way. In a study the Dutch Trimbos Institute [23] concluded that a high average of therapists do use ROM, but mainly the mandatory ROM instrument used for benchmarking by health insurance companies. Therapists do criticize this form of ROM because they indicate that it does not support their clinical practice. The generic nature of the questionnaires and time of measurement at start and end of treatment are points of criticism. The Trimbos Institute describes a gap between the frequency in which ROM is used and the implementation of the outcomes of ROM in treatment. Therapists do not always use ROM as a clinical instrument to steer a treatment’s course. This is the case for individual therapists, but also in team or peer consultation. Therapists request a ROM-system with several requirements [22]; instruments suitable to design and evaluate treatment plans; therapists being trained in interpreting ROM-data and in how to give feedback to patients; good accessibility of ROM-data by therapists; and ROM-data which patients can understand. To support therapists to implement ROM in treatment it is recommended to use disorder specific instruments, at clinical relevant moments in an user-friendly ROM-system [23].

Previous research about therapists’ and patients’ experiences with ROM was mainly focused on self-report questionnaires; questionnaires which are not disorder specific; and ROM experiences of therapists. The listed criticism was also reported among clients with severe BPD and their therapists who found the standard generic ‘Brief Symptom Inventory’ [5] clinically irrelevant. The BPDSI was therefore added at the Viersprong to the Routine Outcome Process. In this qualitative study we focused on experiences of therapists and patients with this disorder specific ROM instrument.

## Methods

### Participants

Participants were patients (*n* = 10) and therapists (*n* = 10) from the Mentalization Based Treatment (MBT) program at ‘de Viersprong’, Amsterdam. De Viersprong is a Dutch institute specialized in psychotherapy for personality disorders.

All included patients were treated in one of the MBT programs for borderline personality disorder (BPD). DSM-IV diagnoses were assessed by trained clinicians using Structured Clinical Interviews for DSM-IV axis I [10, 15] and axis II disorders [9, 26] (SCID I and II). All of the participating patients met the DSM-IV criteria of BPD. Nine were female, mean age at date of interview was 37.5 (range 25–58). Patients where a good representation of all patients in BPD care at the Viersprong Amsterdam at that time with a mean age of 35 and 1 out of 9 patients being male.

All therapists were extensively trained in MBT and working under the MBT Quality system [4]. They had varying degrees of clinical expertise and training: psychiatrist (*n* = 1), master-level psychologists (*n* = 2), therapists with a post-master degree in Healthcare psychology (GZ-psycholoog) (*n* = 4), psychotherapists (*n* = 2) and (post-master) clinical psychologists (being the highest level of clinical psychology training in the Netherlands; *n* = 1). Eight were female. Therapist selection was representative in disciplines, age and gender.

The MBT treatment program consisted of introductory groups (10–12 sessions) [3], treatment (maximum of 18 months) and follow-up treatment (maximum of 18 months). Patients completed one of the three MBT conditions: 1) intensive outpatient treatment (*n* = 5), 2) three day day-treatment (*n* = 1) and 3) five day day-program (*n* = 4). The intensive outpatient treatment included two times a week mentalizing group psychotherapy, weekly individual psychotherapy, patients received socio-therapeutic consults when indicated and medication consults on request of the team and patient. The three day day-treatment included three times a week mentalizing group psychotherapy, weekly individual psychotherapy, weekly art therapy, weekly mentalizing group therapy, weekly writing therapy, patients received socio-therapeutic consults when indicated and medication consults on request of the team and patient. The program ended with a social hour and community meeting. The five day day-treatment included five times a week mentalizing group psychotherapy, weekly individual psychotherapy, twice a week art therapy, weekly mentalizing group therapy, weekly writing therapy, patients received socio-therapeutic consults when indicated and medication consults on request of the team and patient. The program ended with a social hour and community meeting.

### ROM-procedure

ROM at de Viersprong was used to measure treatment outcomes. Measures were taken every three months by a trained treatment-independent research assistant. Patients and therapists were provided a written ROM-report with baseline, next to last assessment, and last assessment outcomes. Therapists were expected to discuss the outcomes with patients and use the outcomes to guide therapy course and duration. All Therapists were trained in interpreting and using ROM-feedback as a standard procedure in their introductory period.

**Fig. 1:**
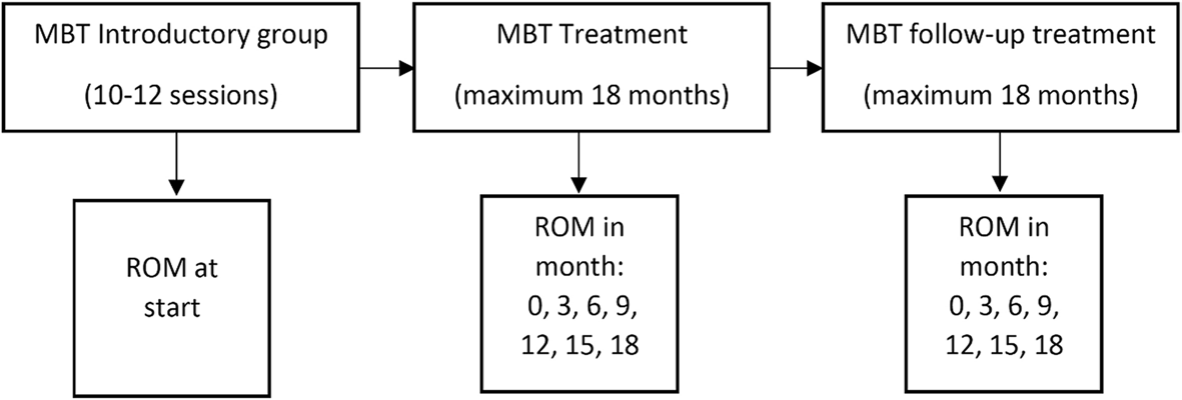
Flowchart of ROM measure moments. Used measures at every ROM measure moment: BPDSI-IV, PDS, BSI and PDS

Measures used at each assessment were the Brief Symptom Inventory (BSI) [5], the Severity Indices of Personality Problems-short form (SIPP-sf) [24], Posttraumatic Diagnostic Scale (PDS) [11] and the Borderline Personality Disorder Severity Index-IV (BPDSI-IV) [1]. Figure 1 presents a flowchart of ROM measure moments.

The BSI [5] is a self-report questionnaire with 53 items, which uses 5-point Likert scales; ranging from 0 (not at all) to 4 (extremely), referring to symptoms experienced in the last week. The BSI measures psychological distress at 9 symptom areas; somatic problems, cognitive problems, interpersonal sensitivity, depression, anxiety, hostility, phobic anxiety, paranoid thoughts, and psychoticism. Besides the symptom areas, the BSI also has the following indices; the mean score of the 9 symptom scales, the quantity of present symptoms and the severity of the present symptoms. The internal consistencies of these scores are reported to be good (Chronbachs alpha ranged from 0.71 to 0.85) [5].

The SIPP-sf [24] is a self-report questionnaire with 60 items, which uses 4-point Likert scales; ranging from 1 (fully disagree) to 4 (fully agree), referring to symptoms in the last 3 months. The SIPP-sf measures 5 domains of personality functioning: self-control, social concordance, identity integration, responsibility and relational functioning. The SIPP-sf is a short version of the SIPP-118. The internal consistencies of these scales are reported to be moderate to good (Cronbach’s alpha ranged from 0.69 to 0.84) [25].

The PDS [11–20] is a self-report questionnaire with 49 items referring to symptoms in the last month. The PDS has four sections. The first section is a checklist which identifies traumatizing events. In the second section the respondent reports which event has been the most upsetting traumatic event. Section 3 assesses the 17 PTSD symptoms based on DSM-IV criteria for PTSD. A 4-point Likert scale is used; ranging from 0 (not at all or only one time) to 3 (5 or more times a week/ almost always). The fourth section assesses the level of impairment of the PTSD symptoms. The PDS yields a severity score from 0 to 51; 0 no rating, 1–10 mild, 11–20 moderate, 21–35 moderate to severe and > 36 severe. The internal consistencies of the total score is reported to be excellent (Cronbach’s alpha = 0.92) [11].

The BPDSI-IV [1] is a semi-structured clinical interview with 70 items to assess the severity of a BPD, referring to the last three months. The items of the BPDSI-IV represent the nine DSM-IV BPD traits. Each of the nine BPD traits is assessed with multiple items. Frequency of the manifestations as described by each item is assessed by an 11-point Likert scale: 0 (never) to 10 (every day). An exception is the ‘identity disturbance’ trait, the items of this scale are assessed on a 5-point Likert scale: 0 (absent) to 4 (dominant) and then multiplied by 2.5. The BPDSI-IV yields a total severity score ranging from 0 to 90. Giesen-Bloo et al. [13] studied the psychometric qualities of the BPDSI-IV and concluded that interrater agreement and internal consistency are excellent. The dysfunctional cut-off score is 14.93, with a high specificity (1.00) and sensitivity (0.97). Giesen-Bloo et al. [14] concluded that reliable change is an improvement of at least 11.70 points.

### Qualitative interview

A semi-structured interview was designed for this study to investigate the experience of therapists and patients with ROM. The therapist-interview contained five topics in the context of ROM: relation between therapist and patient, feedback to patient, ROM and BPDSI-IV in general. An example of a question from the therapist-interview is: *‘Did ROM give you a better insight into the treatment course of your patient? (YES) Can you tell us how you got that insight? (If applicable) Which lists gave you this insight?’* The patient-interview contained five topics in the context of ROM: general questions, relation between therapist and patients, feedback, explanation of therapist about ROM and BPDSI-IV. An example of a question from the patient-interview is: *‘Did the feedback from the ROM provide you with more insight into your symptoms? (YES) What kind of insights did you get? Based on what did you get these insights?’* The full interviews are presented in Additional file 1 (patient interview questions) and Additional file 2 (therapist interview questions).

### Procedure

Patients who had been in MBT treatment were recruited by phone. When patients did not want to participate (*n* = 11), did not pick up the phone (*n* = 20) or the phone number didn’t exist anymore (*n* = 6), the next patient on the list was called. There were three lists of patients, one for every of the three different MBT programs: intensive outpatient treatment (*n* = 34), three day day-treatment (*n* = 10) and five day day-treatment program (*n* = 34). When patients wanted to participate in the study an appointment was made. Appointments were made with twelve patients, there were two no shows. Five patients of the intensive outpatient treatment, one patient of the three day day-treatment and four patients of the five day day-treatment did participate. Participants were given the option to make a face-to-face appointment at de Viersprong (*n* = 4) or an appointment by telephone (*n* = 6). Participants received general information about the nature of the study and the informed consent letter by e-mail. Appointments did not take longer than one hour and participants received 12.50 euros for their participation. Interviews were audio-recorded and transcribed verbatim.

A short introduction about the nature of the study was given to therapists during a MBT meeting at de Viersprong Amsterdam. It was explained that the study goal was to get therapists’ and patients’ experiences to optimize ROM in MBT. All MBT-therapists received an information letter about the study. Afterwards the therapists received an email with a couple of optional dates for an appointment. All therapists entered the study after giving written consent. Appointments did not take longer than one hour and therapists received 12.50 euros for their participation. Appointments with therapists were face-to-face at de Viersprong (*n* = 10). Interviews were audio-recorded and transcribed verbatim.

The study was approved by the ethical committee of the University of Amsterdam (number 2017-CP-8000).

### Analysis

The second author (SC) analyzed the data, based on the semi-structured interviews, thematically. The patient-interviews and the therapist-interviews were analyzed separately. SC read and re-read the transcripts of the interviews to gain familiarity and to gain an overall impression of the collected data. Relevant fragments that seemed to be meaningful were coded and organized in (sub)categories based on the interview topics. Some codes were not distinctive and were merged. SC combined the categories in overarching topics: experiences of therapists and patients with the BPDSI-IV, experiences of therapists and patients with ROM in general and importance of emphasizing ROM culture in organization. A mixed top-down/bottom-up procedure was used to determine the themes. Analyses were conducted in Atlas.ti.

## Results

Table 1 presents the themes and their subcategories that were detected in the material.

**Table 1:** Themes and subcategories

A. Experiences of therapists with the BPDSI	B. Experiences of patients with the BPDSI	C. Experiences of therapists with ROM in general	D. Experiences of patients with ROM in general		E. Importance of emphasizing ROM culture in organization
A1.1 BPDSI as advise for other healthcare organizations A1.2 Only using BPDSI in ROM feedback A1.3 BPDSI as important instrument in treatment A1.4 Familiar with the BPDSI A1.5 Disadvantages of the BPDSI A1.6 Advantages of the BPDSI A1.7 Information meetings about the BPDSI	*B1. Benefits and disadvantages of the BPDSI* B1.1 BPDSI as advise for other healthcare organizations B1.2 BPDSI as standard part of treatment B1.3 BPDSI as important rom instrument B1.4 BPDSI duration B1.5 Insight because of BPDSI B1.6 Disadvantages of BPDSI B1.7 Advantages of BPDSI B1.8 Recommended changes for BPDSI B1.9 Opinions about questions in the BPDSI	*C1. Benefits and disadvantages of ROM* C1.1 Factors to consider C1.2 Increase in ROM outcomes at start treatment C1.3 ROM is alienating C1.4 ROM as valuable C1.5 Disadvantages of ROM C1.6 Advantages of ROM *C2. ROM gives insight* C2.1 Insight because of ROM in combination with clinical view C2.2 ROM insight C2.3 ROM insightful lists *C3. Using ROM in adjusting therapy* C3.1 Using ROM in adjusting therapy C.3.2 Examples of adjusting therapy C3.3 Used lists in adjusting therapy *C4. ROM in evaluation*	*D1. General experiences with ROM* D1.1 General impression D1.2 Recommending the computer ROM lists D1.3 Experiences of computer ROM lists D1.4 point of improvements computer ROM lists D1.5 Experiences with ROM feedback D1.6 Insight because of ROM feedback D1.7 Insightful ROM lists D1.8 Disadvantages of ROM D1.9 Advantages of ROM D1.10 Recommended changes for ROM D1.11 Important for therapist/organization *D2. Effect on therapeutic* *relationship* D2.1 Understanding of therapist D2.2 Experiences with understanding of therapist	*D3. ROM use in evaluation* D3.1 Involvement D3.2 Experiences with ROM feedback D3.3 Insight because of ROM feedback D3.4 Insightful ROM lists D3.5 Linking ROM to evaluation moment D3.6 Linking ROM to choices in adjusting treatment D3.7 Possibilities to ask questions during ROM feedback D3.8 Examples of questions about ROM during ROM feedback D3.9 ROM feedback D3.10 ROM feedback in individual session D3.11 Enough time for ROM feedback	E1.1 Lack of ROM knowledge E1.2 ROM information meetings E1.3 Changing information facilities about ROM E1.4 ROM in science E1.5 Points of improvement for de Viersprong

### BPDSI: benefits

#### Patients

Nine out of ten patients reported at least one benefit of the repeated assessments with the BPDSI. Examples of the various benefits that patients mentioned were: they liked that it specifically measures borderline symptoms (in contrast to the other ROM instruments); that the BPDSI was repeatedly used clarified whether therapy was making a difference; it is exposure to discussing their feelings with someone other than the therapist; that it made them more aware of their symptoms; that it made the symptoms more clear for them; and that it gave them the feeling that the disorder became more clear to the therapist.

“*Well I think it is very good but what I find difficult and certainly found it, especially in the beginning, super confrontational with someone you don't know very well, with whom you are not really going to build an attachment relationship because it is not one of the therapists, to talk about it in that way. Yep, that's just really scary. On the other hand, I think it helped. If I now feel insecure or anxious, it is very difficult for me to use my social environment for this. And my social environment are people that I have selected myself, where I feel confident or not myself. But then when I find that difficult, my mistrust increases and my confidence decreases and I find it more difficult to be vulnerable. Suppose I only had the therapists then I could still think very much 'yes, they are specialists, they will understand me’. And although the person who takes the ROM is of course not just someone off the street, you are kind of forced to talk about it. So I feel it is also a kind of exposure or something and I like that but I also found it difficult.”*

“*The results allowed me to take more account of myself. For example, ‘why is it that my anxiety symptoms have increased?’, then I could reflect on these feelings. That's what I learned here: standing still. Normally, I haven't done that in years. I'm someone who walks away from my feelings. Whatever the outcome was, I always tried to talk to the practitioner about it, about what I had to do, how I can improve.”*

A patient mentioned that from the results of the BPDSI-IV it became clear to the therapist that the patient had self-injured himself, while they had not talked about this subject in therapy. One patient experienced the interview as more personal than filling in a questionnaire (on the computer), because it was possible to explain her answers (note that the BPDSI is a semi-structured interview).

“*In any case, I prefer a conversation more than filling out a questionnaire, it is more personal. Questionnaires are generally the same for everyone. In the conversation you can give your own explanation more, more specifically tell something detailed or something. You can go deeper into it than with a questionnaire. A questionnaire does not really suggest an addition or something. I can never make an addition or add an explanation, I can do that in an interview.”*

Also another patient seems to prefer the approach of the BPDSI interview above the questionnaires on the computer. The interview made her think more consciously about the symptoms she had experienced.

*“Again the interview part because that was what really made me think. You can simply click away those other questionnaires. And you know that at some point if you walk along in psychiatry as a client for a while, then you have had the questionnaires so many times that you can dream them. And actually you think at that moment, ‘it says last week or last three months or last six months’, and then you try to think a bit about it, but you don’t do that very much. While in that interview you do that because it has a completely different approach. And you are much more forced to consciously think about the past week or the past three months.”*

Another patient mentioned that following his own treatment course with the BPDSI, motivated him to go on with treatment.

*“It is a kind of reality check with yourself. You have to give an hour and a half very conscious answers about a recent period and how often something happened. And you find out that one thing still happens more often than you like and the other has actually been reduced a lot. As a result, during the course of the treatment, you will also increasingly see what your own progress is. And that is very motivating to continue with the treatment.”*

Another benefit that was mentioned was that the results made it easier to explain the disorder to loved ones.

*“I can imagine that for other people such insight can also be important for the family, to explain that the therapy goes in a certain way, and I have decreased on this and I have just gotten better on that.”*

To summarize, the above patients gained insight because of the BPDSI-IV.

#### Therapists

Most of the therapists mentioned the disorder specificity as a benefit of the BPDSI-IV; it measured what they were treating the patients for. A therapist mentioned that the subscales of the BPDSI-IV made the symptoms of the disorder more insightful and it made it possible to see changes in treatment course.

*“And then at least you get a little more insight into: ‘hey, what about the different domains, and where are the biggest problems, and what about the emptiness, what about the relationship, what about..?’ “*

Another therapist mentioned that the BPDSI-IV made it possible to classify and that the clear cut-off point made it possible to see if a patient still met the criteria of BPD.

*“So then yes, the BPDSI is what I like to look at, because then you have those subscales matching the BPD features. That those features of the borderline personality disorder are set out over different time periods. Yes, I find that is helpful in the light of: ‘where do I stand regarding my goals and the patient regarding his goals and do I recognize this in the subscales?’”*

Another therapist: *“it does make it very specific, the results on symptom level”.*

### BPDSI: Disadvantages and recommendations

#### Patients

Most experiences of patients with the BPDSI-IV were positive, but seven out of ten also mentioned some disadvantages or recommendations. All but one of these seven patients also mentioned advantages or mentioned that the disadvantages did not outweigh the benefits. Two patients mentioned that the questions of the interview can be hard or difficult i.e. because it was hard to recall how many times things happened and another patient mentioned that sometimes she was done answering questions during the interview, but nevertheless the interview was an important part of the treatment for these three patients.

*“Sometimes you really get tired of it. But after a while you think yes, you know this for yourself.”*

One patient felt as tense as before an exam, however, this did not seem to be as important as the insight the patient gained of himself.

*“The only negative side is more tension for the patient, as a kind of exam; ‘Did I do it well or not?’ More the excitement of ‘what do I hear, how do I do.’ But I don't think that outweighs the image you get about yourself.”*

The same patient also mentioned that he would like to receive the results consequent on paper. Another patient mentioned that some questions about symptoms, which the patient did not recognize at the moment, could lead to certain ideas.

“*Maybe there are also questions between which you normally do not bother and that it gives you ideas or something, that could be. I would say [for] myself, when I am in a very difficult period and there will be a certain question that I had not thought about myself, then it could also give me ideas.”*

The same patient suggested using the interview questions in therapy sessions so they have more time to reflect on the issue.

It was also mentioned that it was confronting to undergo the BPDSI-IV interview with an interviewer which the patient did not know well and without building an attachment relationship. The same patient however explained that you could see this as ‘exposure’, which was a positive experience. One patient mentioned that the questions about addiction and using medication, fears, mood changes and sadness, brought back memories which made her sad and anxious. This patient was wondering if this is positive or negative because it also made her aware of past and current symptoms*.*

*“I get in a mood, of course, that makes me remember things that are not nice, and of course it makes me sad or anxious. But I don't know if it is negative. So it is awareness.”*

The same patient mentioned that a lot of the questions asked of her were no longer relevant which she referred to as a waste of time. For a long time, she was not cutting herself anymore but still it was asked in the interview. She recommended removing irrelevant questions and allocating more time to subjects that are more important to her.

*"Remove the pieces that do not suit me and spend more time on pieces that are more important to me."*

#### Therapists

Two therapists’ said they would like to see a more expanded report of the results of the BPDSI-IV, i.e. with more explanation about the symptoms or a verbatim of the interview*.*

*“Well, the report sometimes may be a bit more extensive. That there is a little more explanation about what exactly the symptoms are. Because then you see, for example, decrease on relationships or on emptiness, but I would like to add a little more words, also for the client. If the client can do something with it. But then you are mainly talking about the report, but I could suggest some improvement there.”*

One therapist mentioned the effort of the patient and interviewer as a disadvantage. Suggested was to take the interview only once every six months instead of once every three months and adding a BPD self-report form in the meantime. Another therapist also mentioned the use of a BPD self-report form which would take less time and would be more easy to use in treatment centers which are not focused on only BPD.

*“It is quite difficult to administer, you have to do an interview. I would really like it if there was a self-report list. Which requires less work, so that it could simply be used much more. Not only here but also elsewhere. Because I think the work where I was before, there was a lot of borderline. But you weren’t just going to do the BPDSI, because I also think that you already suggest a lot. Then you already suggest that someone has borderline. That is possible here because everyone has it when they come here, but in other settings that is much more difficult. So then I would really like it if there was another way.”*

One therapist mentioned the risk of using a semi-structured interview causing important things possibly to be missed because questions can be steered. Another therapist mentioned that the BPDSI-IV is quite literary based on the DSM, the result can be that the questions of the BPDSI-IV can be abstract to the patient, which could cause errors.

*“There was a patient who really had questions like: what is that ‘emptiness’? Then I really had discussions with her, and at a certain point I just took the BPSDI and went to see it with her, and I noticed that these are also quite literal questions about how it is described in the DSM. I think that is a disadvantage, it gives a lot of noise I think. Because it remains so abstract, for example emptiness.”*

Another therapist mentioned that he would like to see more specific description of symptoms accompanying the subscales describing the nature, the frequency and the severity of the borderline symptoms at the time of the measurement.

*‘We already know that we only treat people with borderline. I would like to know more; What do the symptoms look like? How intensive are they? What is the extent of suffering? Has it changed?’*

One therapist questioned the validity of the BPDSI-IV. Two therapists wanted the cut-off point to be less absolute.

*“Clients really can get a terrible tendency: 'yes, but I have to go below 15 because then I will be cured’. That is very sad when the next time is 17, then you have it again. They also become very anxious about it: 'I am now at 14, I have to get out of treatment now because then I no longer have borderline'. So I would like to have it a little less absolute. Also because it can still fluctuate strongly.”*

It was also mentioned that the BPDSI-IV only measures the DSM- personality characteristics and that it does not measure the level of personality functioning.

*“So you measure the outcomes, the consequences of the personality problem, but you don't measure the personality problem in itself. So that's a disadvantage. But I understand very well that it goes like this. We also do a SCID in the intake here. You indicate someone here for treatment based on their symptoms. So I also understand that you measure it that way, but that is of course really a shortcoming. That you don't, understand how someone is as a person, and how things are going differently. You only measure the characteristics.”*

## Experiences of therapists and patients with ROM in general

### Importance and use of ROM feedback

#### Patients

ROM feedback was usually given to patients during evaluations in which treatment plans were being discussed. Some therapists’ gave ROM feedback during an individual session (before an evaluation). Patients mentioned that ROM feedback was used to make decisions in treatment, like formulating treatment goals or transition to aftercare and to monitor the treatment course.

“*I work in education, I teach, but I also want there to be a learning track and that we work towards something and that you can hold on to something that you can test. Yes I don't know. For me it makes perfect sense in the learning process that you test and monitor. And that you report results and you look back on it, I don't know. In my head that cannot exist without each other.”*

*“I would prefer to always be in therapy, that is just very safe and you have a place where you can discuss everything. In the beginning it is very difficult to find that trust. And once you have that trust, it is very difficult to let it go and do it by yourself. I think it is very nice for both the therapist and the client that you have that ROM to say: ‘Yes, it is going well, so you can do it’. The moment you didn't have the ROM, it might just be based on the conversations. Now you really review exactly what happened in the past three months.”*

Most participants gained insight in their disorder because of the ROM feedback. It gave insight into their own suicidality, the treatment course, the kind and the severity of the symptoms.

*One patient describes it as “A piece of awareness. You are by no means aware of everything if it is not mentioned; that something goes better or worse.”*

Not all participants got a better insight in or monitoring of their symptoms through ROM, for example one participant mentioned a lack of confidence in ROM and another participant had a comorbid disorder which made ROM confusing.

Four patients felt the therapists got a better understanding of the patient because of ROM. This was mentioned by two patients who found it hard to explain emotions or symptoms, one patient who had varying ROM results which was discussed in treatment, and one patient who felt that ROM brought up subjects which aren’t discussed in therapy sessions.

*“Of course, a lot of questions are asked during ROM. Questions that may not normally surface during a conversation. So that you address other topics than you would just have in a conversation.”*

#### Therapists

” *There is a perverse incentive behind ROM.”*

ROM was described by one therapist as initiated by and as an obligation of the insurance company. Money is invested in ROM but could also be spent on better care. However, that did not seem to be the dominant picture of ROM among the participating therapists.

*A therapist described ROM as: "ROM is actually just the figure with which you say: ‘where are we actually regarding this goal’. It is also very much about what we have actually achieved here in the recent period.”*

ROM was described as a *'quick screener'* for present psychiatric symptoms, which can be discussed with the patient. ROM is seen as an addition to the previously formulated treatment goals and measuring every three months ensures that there are differences in symptoms over time.

*Another positive side effect of the ROM is described as: ‘An instrument that objectively goes through all those complaints and scales, it is an addition to your clinical view’.*

*Another therapist gave the following example: “You work here in attachment relationships where transference plays a major role and your view can be clouded. If all goes well, this kind of measurement provides you with a clear view.”*

It was experienced as pleasant that someone from outside the team, the test psychologist, was engaged. This, because the test psychologist is not directly involved in the dynamic of the patient and therapist. In addition, the patient is involved in the treatment by ROM.

Therapists’ however also were critical about ROM and do question the reliability of ROM. It was frequently mentioned that ROM couldn’t be seen separately from clinical observations by the therapist. It was also mentioned that ROM is a snapshot, depending on situational circumstances.

*“For example, sometimes you come by a client whose ROM outcomes deteriorate greatly in the first few months, but sometimes that is not a bad sign at all. Because someone starts to feel more and think about these feelings more. In that case it is true the symptoms increase but then it would be misinterpreting ROM when one would say it is not going well. So in that sense I think the displayed outcomes are on a superficial level. So I just think ROM is not enough to say anything about the treatment result because my clinical view is also absolutely essential.”*

ROM was described as expensive and time-intensive. At de Viersprong, four ROM instruments were taken, while one is the most used in therapy, namely the BPDSI-IV. A therapist wonders whether the cost benefits of the ROM would be positive.

*Another therapist: ‘Many of those questionnaires are self-report lists and they are not reliable in people with borderline. Some people can really display something quite well about how things are going now or how things have been going over the past period. Some people can't. The weaker people are structured, the more distortions there are sometimes. I think that is really a problem with these measurements.’*

One therapist mentioned that there might be a learning effect among patients in answering ROM questionnaires, a coloring of answers due to not wanting to complete the treatment or, on the contrary, giving ‘better’ answers than the patient actually feels, or choosing a good day for filling out ROM. Patients may see the ROM as a school report or experience the feeling of having to perform. Therapists wonder if you can measure the effect of the treatment based on symptoms.

” *So then the experience of the patient becomes a piece of paper. The experience is externalized and the patient has to read on a piece of paper whether he is doing better and I find that very strange, I find that surprising. Because it assumes that we can measure it very well and that we therefore assume that it is a result of treatment.”*

Other shortcomings mentioned are possible bias, too many confounders, and a number of organizational shortcomings such as the planning of ROM.

## Importance of emphasizing ROM culture in organization

### ROM education for therapists

Therapists have different experiences with education about ROM. A therapist mentioned he hadn’t had any education about ROM and another therapist couldn’t remember having received education about ROM. Another therapist mentioned that therapists have undoubtedly been given information but was unable to remember this well. Three other participants also mentioned that education about ROM was a long time ago. Four participants mentioned that they had received an explanation about ROM from the test psychologist during their introduction period.

### ROM culture among therapists

It is remarkable that more than half of the therapists mentioned that they mainly used the BPDSI-IV in treatment and that they choose to not use the other lists because of various reasons. For example, a therapists mentioned that the PDS is too sensitive; two therapists mentioned that the PDS was not applicable for the patients; another therapist mentioned that the BSI always yielded high scores; and four therapists mentioned that they did not use (all) the instruments optimally due to a lack of knowledge. Examples of a lack of knowledge are therapists who are not sure how to explain or provide feedback about the lists, just recently found out that the BPDSI-IV is an interview, do not know how to integrate the lists together and neither how to integrate the lists in therapy, or are not familiar with the content of the lists. Therapists would like to receive more information about ROM, so they can use the ROM more optimally. There also seemed to be a need for knowledge about the developments of ROM with regard to the insurance companies, so they can explain this to the patient. Therapists were thinking about education about ROM in different types; brochures that are easy to copy, a refresher course every now and then, but also feedback about the aggregated ROM results of the department. A therapist also expressed a need for a guideline for feedback and interpretation of ROM:

“*Because now we do it a bit because it has to be done, and I can give it a twist, but that is really in my own way. Everyone would have a different way, so that doesn't seem very good to me either.”*

A good implementation of ROM was seen as important. On the one hand to motivate patients for ROM, on the other hand to make therapists feel familiar with ROM.

*"I think when you can see what it gives you it is helping, but if you can’t then it becomes a [sigh] you know, then it just becomes something you have to do extra and you would think something like: ‘I am already telling to my therapist how it goes?’ And if you don't make that clear, it just becomes an annoying list. So yes, that is not actually my opinion, I think it can really have an added value, but you would also need a mutual culture to consider it in that way and the substantive added value is also visible.”*

Another therapist mentioned the importance of training new employees in ROM in order to emphasize the importance of ROM in the organization.

## Discussion

In this study we found that in general experiences of patients and therapists whit the BPDSI-IV were positive and the use of a disorder specific ROM instrument seems promising and valuable.

As was found in other studies [7–23], therapists for different reasons sometimes found it hard to use ROM-output in their evaluation.

Patients and therapists seemed to prefer the BPDSI-IV above the other self-report ROM instruments. Patients mainly because it reflected their symptoms best, for therapists this was an important argument as well, but another reason was they did not know how to interpret the other instruments. This is remarkable since guidelines and knowledge about the use of ROM was available at de Viersprong; there were ROM workshops, there were flashcards about ROM, and the tests psychologists are there for uncertainties about ROM. Besides the possibilities about gaining information about ROM, the organization attempted to implement a strong ROM-culture; patients received ROM every three months via e-mail, patients were able to sign themselves up for the BPDSI-IV interview, therapists received monthly reminders via e-mail, ROM was part of the training program of new therapists, and it was part of the Quality System of MBT. Despite the many given possibilities for effective use of ROM, therapists do not seem to recognize a strong ROM-culture.

A strong culture in using quality systems in organizations is important as it decreases the variation between therapists in use [12]. Despite the attempted ROM-implementation and ROM-organization culture at de Viersprong, therapists did question the utility of ROM and did not all feel facilitated in ROM-use. As was found in the study of van Geffen [12] across different therapists across different organizations, MBT therapists interviewed in this study also seem to choose which quality system(s) they use based on their personal preferences. Van Geffen [12] interviewed therapists about their feedback propensity since this seemed to be a determining factor in deciding to use ROM or not. In the van Geffen study many therapists mentioned that the measurements of the systems were not reliable because patients just answered something; scores were easy to manipulate; measures were just snapshots; self-reporting gives a distorted view; or the data in the systems were incorrect. Many therapists had more trust in their own clinical view than in quality system feedback [12]. The MBT-therapists responses in this study fit this profile exactly. MBT-therapists made exceptions for the BPDSI-IV since it was disorder specific. These findings are important because an open attitude of the therapist towards feedback and a high self-efficacy of the therapist is known to have a positive influence on treatment progress [7]. Therapists who are not using ROM effectively can be therapists with a lack of progress in treatment.

Even in a ‘ROM-valuing organizational context’ ‘the doctor knows best principle’, mistrust pertaining to the incorrect use of ROM-data by insurance agencies or the organization, and the idea of patients not being capable of reporting their symptoms objectively and to the full extent runs freely among therapists. The contrast with patients experiencing ROM as helpful for self-esteem, treatment monitoring, and enhancing and shortening the treatment process is disturbing and in contrast with scientific outcome [19–22]. The ability to receive and use feedback, therapists’ self-esteem and the capacity of self-doubt might be important therapist factors influencing the effective use of ROM as well as shared decision making. Future studies could focus on these and other possible therapists’ factors influencing use of ROM specifically and treatment outcome generally.

One needs to be careful with generalizing the results of this study to other patients and therapists. Limitations of this study are that both groups were of a small sample and both groups were part of one treatment center and of one specific treatment program (MBT). However, Guest, Bunch and Johnson [16] researched how many interviews were enough to reach saturation and concluded that within the first twelve interviews saturation occurred and meta-themes were reached in six interviews.

The present study was conducted among patients who received MBT and among MBT-therapists. As type of therapy might be of influence, future studies should study patients’ and therapists’ views of different forms of psychotherapy.

Although the patient and therapist samples where representative for both groups within the Viersprong, there where few men in both samples. Future research can shed light on the question whether there is any gender difference in the subjective experiences with Routine Outcome Monitoring. Other limitations of this study are that there was no member-check and analyses weren’t double classified by a second rater, so it wasn’t possible to calculate the inter-coder agreement. To limit this negative effect the coder was well supervised by the first author, hence the coding was not a pure individual-subjective activity. The fact that the study was unfunded limited our possibility to employ multiple coders.

Since the BPDSI-IV needs training for interviewers and takes approximately 45–60 min per interview, future studies could compare the BPDSI-IV to other BPD-specific or a more generic Personality symptom self-report questionnaire such as the Severity Indices of Personality Problems (SIPP-118) [25] to investigate which instrument is preferable for clinical implementation.

Although the aim of the present study was to investigate how a BPD-specific outcome measure was evaluated by stakeholders, also in comparison to a generic psychopathological symptom outcome, the study did not address how an outcome assessing functioning would be valued. An important topic for future studies is to investigate how an outcome assessing functioning is evaluated, also in comparison to a measure assessing disorder-specific symptoms.

## Conclusions

Organizations could improve or extend their ROM-culture not only focusing on a facilitated ROM-process, but also to train and inform therapists about how to give ROM feedback, how to interpret ROM, how to combine ROM with clinical impressions (which indeed are important) and how ROM is exactly used within the organization and in relationship to insurance agencies. It also seems to be important to support therapist self-esteem and stimulate them to discuss ROM-output in their professional team and peer-supervision to overcome the (sometimes difficult to bear) idea the therapist did not improve the patient and open up the option a treatment does not fit, or the therapist might not fit the patient or the treatment. Mental health care can profit from a more open culture where making mistakes, or not being a good therapist (in a specific context) is an option. This will increase learning possibilities and expectedly decrease the number of unsuccessful treatments.

As was found in this study [12], the present study found that some therapists did not take their patients’ perspective seriously which was a reason not to use ROM-outcome to guide the treatment process. This points to another important issue in mental health care to facilitate ROM might not only be a ROM culture, or a culture of shared decision making, but a culture shift on patient image where organizational culture would be characterized by equality between professionals and patients. With the difference that professionals are therapy experts however patients are experts of their lives and symptoms. Future studies might focus on influencing professional stuck points on patient image to improve ROM- and treatment outcome.

## Supplementary Information

**Additional file 1.**Interview patients.

**Additional file 2.**Interview therapists..

## Data Availability

The datasets used and/or analysed during the current study are available from the corresponding author on reasonable request.

## Abbreviations

BPDSI-IV: Borderline Personality Disorder Index-IV
BSI: Brief Symptom Inventory
MBT: Mentalization Based Treatment
PDS: Posttraumatic Diagnostic Scale
ROM: Routine Outcome Monitoring
SIPP-sf: Severity Indices of Personality Problems-short form
SCID I and II: Structured Clinical Interviews for DSM-IV axis I axis II disorders
