# Supplementary material for: The use of borderline personality disorder severity index-iv feedback in adjusting borderline personality disorder treatment: therapists and patients perspectives
Source: BMC Psychiatry. 2022 Jul 14;22:469. doi: 10.1186/s12888-022-04104-w (PMC9284892; doi:10.1186/s12888-022-04104-w)
Supplement: Supplementary file 1 — Additional file 1.Interview patients. [file 12888_2022_4104_MOESM1_ESM.docx]

**Additional file 1: Interview patients**

*General questions*

1. Can you tell us what your life is like right now (work, social, family)?

2. Are you currently in treatment?

1. (YES) For which complaints? What kind of treatment?

*Relationship with Therapist*

3. Did ROM helped your therapist in understanding you?

1. (YES) How did you notice that?
2. (NO) What do you think about that?

4. (If applicable) Which ROM list do you think was the result of your therapist understanding you better?

*Feedback*

5. Did you get feedback of the ROM results?

1. (YES) How did that go?
2. (NO) What do you think about that?

6. Did you have the opportunity to ask questions during the feedback?

1. (YES) What kind of questions did you ask?
2. (NO) What do you think about that?

7. Did the feedback from the ROM provide you of more insight into your symptoms?

1. (YES) What kind of insights did you get? Based on what did you get these insights?

8. (If applicable) Which questionnaires gave you this insight?

9. Were the results of your ROM linked to your treatment evaluation and choices in your treatment (for example, transition to aftercare, making a reduction schedule etc.)?

1. (YES) For which lists did this apply?

10. Did you feel that the therapist made enough time for feedback of ROM?

1. (YES) How did you notice that?
2. (NO) What do you think about that?

11. Did you feel that you were more involved in your treatment process because of ROM and feedback?

1. (YES) how did you notice?
2. (NO) And what do you think about that?

12. (If applicable) For which questionnaires did this apply?

*Therapist's explanation of ROM*

13. Explained the researcher / therapist what the process of ROM would look like during treatment?

14. Did the researcher / therapist explain what the purpose of the ROM was?

a. (YES) Could you describe this goal?

*BPDSI in particular*

15. What do you think of the questions of the BPDSI?

16. What do you think of the duration of the BPDSI?

17. If you could change one thing about the BPDSI, what would it be?

18. Do you consider the BPDSI as ROM as an important part of treatment and would you advise making this a regular part? Why?

19. Are there any negative / positive sides of the BPDSI that offered you personal insight?
