# Supplementary material for: The use of borderline personality disorder severity index-iv feedback in adjusting borderline personality disorder treatment: therapists and patients perspectives
Source: BMC Psychiatry. 2022 Jul 14;22:469. doi: 10.1186/s12888-022-04104-w (PMC9284892; doi:10.1186/s12888-022-04104-w)
Supplement: Supplementary file 2 — Additional file 2.Interview therapists.. [file 12888_2022_4104_MOESM2_ESM.docx]

**Additional file 2: Interview therapists**

*Relationship with patient*

1. Did ROM gave you a better insight into the treatment course of your patient?

1. (YES) Can you tell us how you got that insight?
2. (If applicable) Which lists gave you this insight?

2. Did ROM made you understand your patient better?

1. (YES) How did ROM manage this? How did you notice that?
2. Which lists provided this?

3. Do you use the results of ROM to adjust the treatment? Is that: Never - Rarely - Sometimes - Regularly - Often – Always

1. When will you do it? Why?
   - Which lists did you used for this?
2. When don't you do it? Why not?

*Feedback to patient*

4. How often do you give ROM feedback to the patient? Is that: Never - Rarely - Sometimes - Regularly - Often – Always

1. When will you do it? Why?
   - About which lists do you give feedback?
   - Can you tell what that feedback looks like?
2. d) When don't you do it? Why not?

5. Do you discuss the results of ROM with the researcher who has took the lists? Is that: Never - Rarely - Sometimes - Regularly - Often - Always

1. When do you do this? Why?
   - How is this conversation going?
   - Which lists do you discuss? Why this one?
2. When don't you do it? Why not?

6. Do you have the feeling that you do have enough time for ROM feedback to the patient?

1. (YES) How did this turn out?
2. (NO) Why not?

7. Has the patient the opportunity to respond during feedback?

1. (YES) How was the patient allowed to do this?
2. (NO) Why not?

*BPDSI and ROM in general*

8. Are the results of patients on ROM discussed in the team?

1. In what way?
2. What do you think about that?

9. Can you describe how you have been informed about ROM?

1. What did you think of that? Would you like to see this differently?

10. Could you tell us a bit more about your thoughts on ROM?

1. What are the advantages / strengths?
2. What are the disadvantages / weaknesses?

11. If you could leave out a list from ROM which one would it be? Why?

12. If you could add a list to ROM what would it be? Why?

13. Could you describe how you have been informed about the BPDSI?

1. What do you think of that? Would you like to see this differently?

14. Could you tell us a bit more about your thoughts on the BPDSI?

1. What are the advantages / strengths?
2. What are the disadvantages / weaknesses?

15. If you could change one thing about the BPDSI, what would it be? Why?

16. Do you consider the BPDSI as ROM instrument as an important part of treatment and

would you advise making this a regular part? Why?
